# X chromosome drive is constrained by sexual selection and influences ornament evolution

**DOI:** 10.1098/rspb.2023.0929

**Published:** 2023-07-26

**Authors:** Kimberly A. Paczolt, Gabrielle T. Welsh, Gerald S. Wilkinson

**Affiliations:** Department of Biology, University of Maryland at College Park, College Park, MD 20742, USA

**Keywords:** stalk-eyed fly, meiotic drive, multiple mating, diopsids, experimental evolution

## Abstract

Experimental evolution provides an integrative method for revealing complex interactions among evolutionary processes. One such interaction involves sex-linked selfish genetic elements and sexual selection. X-linked segregation distorters, a type of selfish genetic element, influence sperm transmission to increase in frequency and consequently alter the population sex ratio and the opportunity for sexual selection, while sexual selection may impact the spread of X-linked distorters. Here we manipulated sexual selection by controlling female mating opportunities and the presence of a distorting X chromosome in experimental lines of the stalk-eyed fly, *Teleopsis dalmanni*, over 11 generations*.* We find that removal of sexual selection leads to an increase in the frequency of the X-linked distorter and sex ratio across generations and that post-copulatory sexual selection alone is sufficient to limit the frequency of distorters. In addition, we find that male eyestalk length, a trait under pre-copulatory sexual selection, evolves in response to changes in the strength of sexual selection with the magnitude of the response dependent on X chromosome type and the frequency of distorting X chromosomes. These results reveal how a selfish X can interact with sexual selection to influence the evolution of sexually selected traits in multiple ways.

## 1. Introduction

The evolutionary consequences of sexual selection can be influenced by demographic changes caused by environmental or intrinsic factors, such as a distorting sex chromosome [1]. X-linked segregation distorters, also known as X-linked meiotic drivers, are selfish genetic elements that bias their transmission by interfering with the development of Y-bearing sperm. Males bearing these elements produce more daughters than expected under Mendelian segregation, causing a *sex ratio* (SR) phenotype [2–4]. As an X-linked distorter increases in frequency, the population is expected to grow [5] with the sex ratio becoming increasingly female-biased and, in the absence of opposing forces, can result in rapid population decline or extinction due to sperm limitation caused by male rarity [1]. Such changes in the population sex ratio alter the opportunity for and intensity of sexual selection [6].

Post-copulatory sexual selection can limit the spread of selfish elements, such as X-linked distorters, when females mate with multiple males [4]. SR males produce half as many mature sperm from each spermatocyst as compared to standard (ST) males and consequently are expected to be sperm-limited sooner than ST males when the population sex ratio is female-biased [7], unless spermatocyst production increases to compensate [8]. Post-copulatory competitive success of SR males may also be reduced if SR ejaculates are inferior to ST ejaculates [9,10] or if females are able to bias fertilization against SR males [10,11].

Pre-copulatory sexual selection may also oppose the spread of X-linked distorters. Associations between X-linked distorters and mating success [12] or between X-linked distorters and pre-copulatory traits preferred by females [6,13] have been documented in a few species, although examples are rare [14]. For pre-copulatory sexual selection to act against a segregation distorter, female preference should favour an honest indicator of the male's genotype [15], which is more likely to evolve in cases where low or no recombination maintains strong linkage between the indicator and drive loci, as can be caused by multiple overlapping chromosomal inversions [6].

The stalk-eyed fly, *Teleopsis dalmanni* (*species 1*; see [16]), provides an ideal system for determining how sexual selection interacts with an X-linked distorter because male traits under sexual selection are influenced by SR status. At dusk, males compete to join groups of females, and most mating occurs within these groups shortly after dawn [17,18]. Eyestalk length is an allometric, condition-dependent ornament [19,20], where males bearing long eyestalks experience an advantage in both female choice and male–male competition [21–23]. SR males have shorter eyestalks relative to body size when compared to ST males [13,24] and should thus be at a disadvantage in pre-copulatory sexual selection, which is expected to contribute to maintenance of the drive polymorphism [6]. Several lines of evidence [25,26] indicate that post-copulatory sexual selection is also important in this species. Both sexes will remate multiple times each day [21]. Success in sperm competition assays is variable [27], perhaps because often fewer than 100 sperm are transferred in a spermatophore [26,28]. SR males have shorter sperm than ST males [29], and SR males are less successful in gaining fertilizations when competing against ST males [30]. However, testes of SR males grow faster than ST males in apparent compensation for reduced sperm production [8,31].

Male *T. dalmanni* with the SR phenotype produce 94% female offspring, on average, due to a distorting X chromosome (X^SR^) that fails to recombine with the non-distorting X chromosome (X^ST^) [16] as a consequence of a series of overlapping inversions [32]. The X^SR^ chromosome is highly diverged from and has less genetic variation than the X^ST^ chromosome [32,33]. The X^SR^ chromosome decreases egg-to-adult survival for both males and females [34], although X chromosome type does not impact adult survival [30]. Adult females of all three X chromosomal combinations are fertile, and X^SR^X^ST^ females have a fecundity advantage [30].

Here we use experimental evolution [35] to determine the consequences of sexual selection on the spread of an X-linked distorter by manipulating the number of mates available to females in the presence or absence of an X^SR^ chromosome. In contrast with imposing artificial selection on a single trait, experimental evolution allows for the evaluation of hypotheses in which more than one selective force is suspected. Given that either pre- or post-copulatory sexual selection could limit the spread of X^SR^, we created treatments in which both pre- and post-copulatory, primarily post-copulatory, or no sexual selection could occur. We predicted that the manipulation of sexual selection would result in a change in X^SR^ frequency between treatments with subsequent effects on sex ratio and fecundity within lines, and that these differences could further impact the intensity of selection. Therefore, we measured relative eyespan as a target of pre-copulatory sexual selection [18,22] and expected that prevention of pre-copulatory sexual selection would lead to a reduction in male eyespan.

## 2. Methods

**Figure 1.**
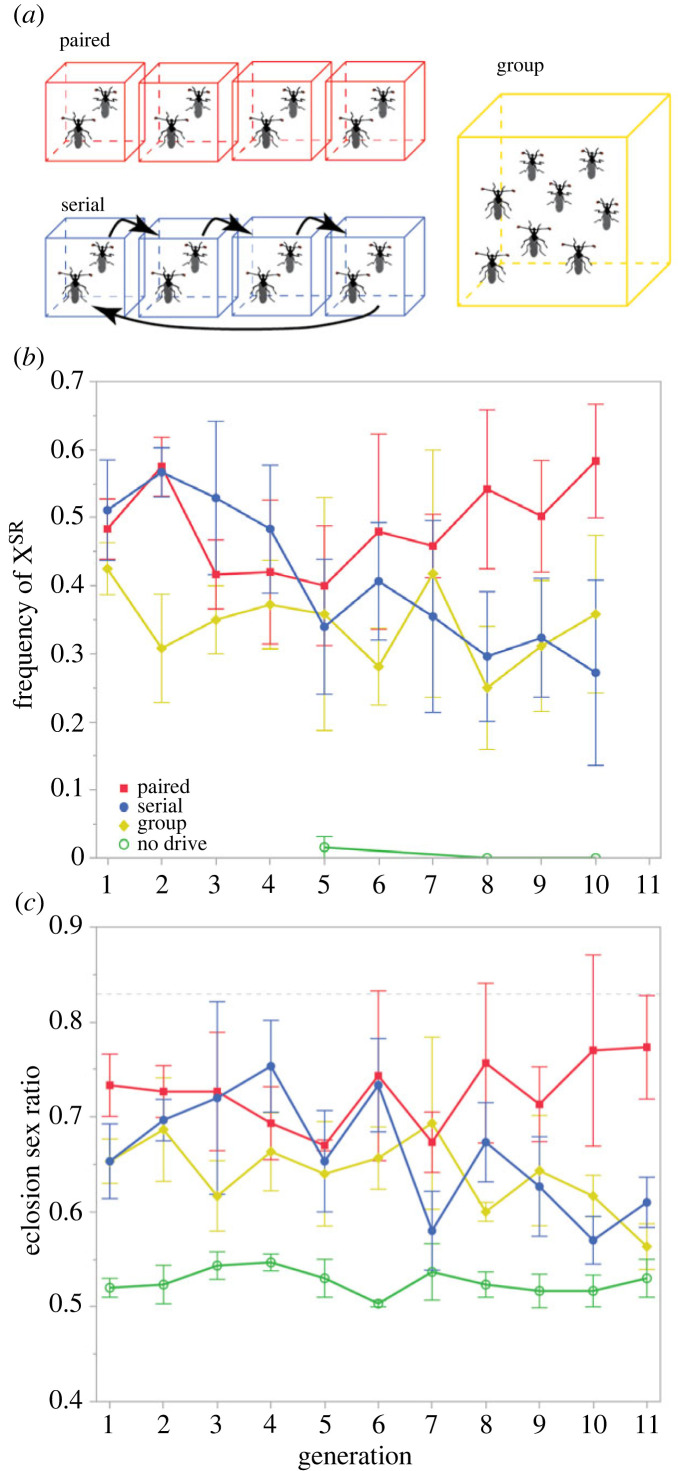
(*a*) Female mating opportunities were controlled during a four-day mating period in three replicates of each treatment. Females had access to one male in the paired treatment (red), four males (one per day) in the serial treatment (blue) and all males in the group treatment (yellow with drive, green without drive). The sex ratio among the 60 breeders varied to match each generation's sex ratio at eclosion. Mean (s.e.m.) (*b*) frequency of X^SR^ chromosomes and (*c*) sex ratio at eclosion over time for paired, serial and group treatments with drive, and group treatment with no drive. At the end of the experiment, the frequency of X^SR^ and eclosion sex ratio were higher in the paired treatment, while serial and group treatments were similar to starting conditions. Whenever eclosion sex ratios exceeded 83% (*b*, dashed line), breeding sex ratio was fixed at 10 males and 50 females.

To assess the consequences of pre- and post-copulatory sexual selection on X^SR^ dynamics, we created three replicate lines for each of three treatments in which female mating opportunity was manipulated during a four-day mating period after flies had attained sexual maturity at three weeks of age (figure 1*a*). Each line was maintained for 11 nonoverlapping generations. In the first generation of the *serial* treatment, 30 females were each sequentially housed with four of 30 males, one per day over four days. Sexually mature *T. dalmanni* females typically mate several times per hour each day [36], so this treatment permits multiple mating, and consequently post-copulatory sexual selection, while limiting pre-copulatory sexual selection. In the first generation of the *group* treatment, 30 males and 30 females were group-housed to allow both pre- and post-copulatory sexual selection. Finally, in the first generation of the *paired* treatment, 30 females were paired with 30 males for four days to prevent pre- or post-copulatory sexual selection. The lines for these three *drive* treatments were started with an X^SR^ chromosome frequency of approximately 30%, which is similar to or higher than that of wild populations [16,36,37]. Drive frequency was manipulated using flies of known X chromosome type (see electronic supplementary material for details).

To determine if the presence of X^SR^ chromosomes and subsequent alteration of the breeding sex ratio affects the evolution of sexually selected traits, we also established three replicate lines of a fourth treatment (*group, no drive*), which lacked detectable X^SR^ chromosomes. These lines were maintained by group mating.

To incorporate the effects of drive on breeding sex ratio in each line, subsequent generations were created by selecting 60 breeders from pools of mature virgin flies to match the sex ratio at eclosion of that line in that generation. The sex ratio among breeders, therefore, became female-biased when males carrying X^SR^ chromosomes were present. Consequently, males in the paired or serial treatments were housed with more than one female at a time when the drive chromosome increased in frequency (electronic supplementary material). Moreover, when the eclosion sex ratio deviated from 1 : 1, the intensity of both pre- and post-copulatory sexual selection on male traits in each line could vary in all mating treatments. To prevent line extinctions and ensure flies from each line could be genotyped and phenotyped at the end of the experiment, breeding sex ratio was capped at 83% females, i.e. each line always had at least 10 breeding males.

After the four-day mating period, males were removed and all breeding females in each line were housed together for four weeks while eggs were collected (electronic supplementary material, figure S2); collections after week four had no pupae, indicating that females had exhausted their viable stored sperm (electronic supplementary material). Offspring eclosing from eggs laid in the first two weeks were counted and sexed; these values were used to measure eclosion sex ratio as the proportion of female offspring at each generation and the average number of offspring produced by each female (electronic supplementary material). Flies were kept as virgins for at least four weeks to attain reproductive maturity before mating. Feeding and housing were standardized across the lines (electronic supplementary material) when not mating to minimize effects of larval and adult crowding [38]. Eyespan, body length and thorax width were measured to the nearest 0.01 mm for all mated flies in each generation [39].

The frequency of X^SR^ was estimated in each drive line every generation by genotyping 20 mated females (i.e. sampling 40 X chromosomes) with two drive-diagnostic markers; a microsatellite, MS395 [16,40], and an indel (electronic supplementary material). We genotyped all mated flies in all lines at generations 0, 5 and 10, which confirmed that the frequency of X^SR^ was absent or very low (0–4%) in the *group, no drive* lines throughout the experiment (figure 1*c*).

To identify factors that influenced the experimental evolution outcomes we fit linear mixed models (LMMs) with REML using JMP Pro v15.2.0 [41] and report F statistics (*F*) and *p*-values for each effect. We report the estimate ± s.e. for each random effect and a Wald *p*-value evaluating whether the estimate differs from zero. *Post hoc* tests were completed using either a Student's *t*-test to compare levels of direct effects or analysis of means (ANOM) for interactions to evaluate whether each group differs from the overall mean.

To predict eclosion sex ratio, expressed as the proportion of female offspring, and X^SR^ frequency over time, we fit LMMs with treatment, generation and their interaction as independent variables, and included line nested within treatment as a random effect. We excluded the initial generation when fitting these models because we imposed the starting sex ratio and drive frequency, whereas subsequent eclosion sex ratios were a consequence of the frequency of drive-carrying males in each line. To determine if mating treatment influenced female reproductive success in drive lines, we fit a LMM on offspring production per female in drive lines, which may be affected by both the number of eggs a female can lay and the availability of viable sperm. This model included treatment, generation, the interaction between treatment and generation, sex ratio among breeding individuals, and the average length of breeding females in each generation of each line (*N*_total_ = 3616) as independent variables, with line nested within treatment as a random effect. A LMM on offspring production per female in group-mated lines was used to assess whether the presence of drive chromosomes impacted per female productivity. Effects of treatment, generation, their interaction and average female length were included, with line nested within treatment as a random effect (electronic supplementary material). We also fit a LMM on offspring production per line to test the expectation that population productivity is highest when SR causes moderately female-biased sex ratios. This model included treatment, generation and their interaction, breeding sex ratio and the square of breeding sex ratio as independent variables and replicate nested within treatment as a random effect (electronic supplementary material).

To determine if the mating treatment influenced evolution of a trait under pre-copulatory sexual selection [21,22], we analysed eyespan and body length from 634 genotyped males and 393 genotyped females at the end of the experiment. To ensure we had sufficient flies of each X chromosome type from each line, we included flies measured and genotyped in generations 10 and 11 but excluded three male and four female outliers identified by a linear regression between eyespan and body length (males *R*^2^ = 0.72, *p* < 0.0001; females *R*^2^ = 0.70, *p* < 0.0001). We first analysed whether either male or female body size differed between treatments at the end of the experiment with a LMM that included treatment, X chromosome type, their interaction and a random effect of replicate nested within treatment (electronic supplementary material). We then predicted relative eyespan of each sex with LMMs that included body size as a covariate, treatment, X chromosome type and all interactions between these variables and body length, and random effects for line nested within treatment and generation (10 or 11). We also calculated ordinary least-squares (OLS) slopes to describe the allometric relationship [42] between eyespan and body size for males of each line and X chromosome type and females of each line. Female OLS slope was not calculated separately for each X genotype because we detected no effect of X genotype on female relative eyespan (table 3). We compared slopes in males using a linear model with treatment, X chromosome type, average breeding sex ratio and the interaction between X chromosome type and average breeding sex ratio, calculated as the arithmetic mean over generations 1–10, as factors. We included average breeding sex ratio to evaluate its possible impact on selection intensity within each line.

## 3. Results

As predicted, removing pre- or post-mating sexual selection in the presence of a driving X chromosome resulted in pronounced differences in X^SR^ frequency and eclosion sex ratio over 11 generations of experimental evolution. Relative to the starting conditions (sex ratio = 0.5; mean frequency of X^SR^ = 0.325, range = 0.275, 0.375), frequency of X^SR^ and eclosion sex ratio initially increased in all drive-carrying lines but after the first generation the mating treatments began to diverge (figure 1). Fitting a LMM to frequency of X^SR^ revealed a significant interaction between treatment and generation (table 1; electronic supplementary material, tables S1 and S2). The interaction is due to the frequency of X^SR^ increasing over time in the paired treatment (figure 1*b*; *post hoc* ANOM, *t* = 2.63, *p* < 0.01), as expected when sexual selection is removed, while it declined in the other two drive treatments. The LMM on eclosion sex ratio also revealed an interaction between treatment and generation (table 1; electronic supplementary material, tables S3 and S4). As expected, the higher frequency of the X^SR^ chromosome in the paired treatment lines resulted in increasingly female-biased eclosion sex ratios over time (figure 1*c*; *post hoc* ANOM, *t*_paired_ = 2.19, *p* < 0.05). By contrast, eclosion sex ratios declined in both the serial and group treatments (*post hoc* ANOM, *t*_serial_ = −1.68, *t*_group_ = −0.51). In both models, the random effect of line did not differ from zero (frequency of X^SR^: 0.016 ± 0.010, *p* = 0.10; eclosion sex ratio: 0.003 ± 0.002, *p* = 0.1432).

**Table 1.** Results from LMMs on frequency of X^SR^ (f(X^SR^)) and sex ratio in *drive* lines, where line is a random effect nested within treatment, and treatment, generation and their interaction are fixed effects.

effect	f(X^SR^)			sex ratio		
	d.f.	*F*	*p*	d.f.	*F*	*p*
mating treatment	2, 6	0.86	0.47	2, 6	1.93	0.22
generation	1, 78	7.12	0.0093	1, 87	3.85	0.053
treatment * generation	1, 78	8.93	0.0003	2, 87	3.93	0.0233

**Figure 2.**
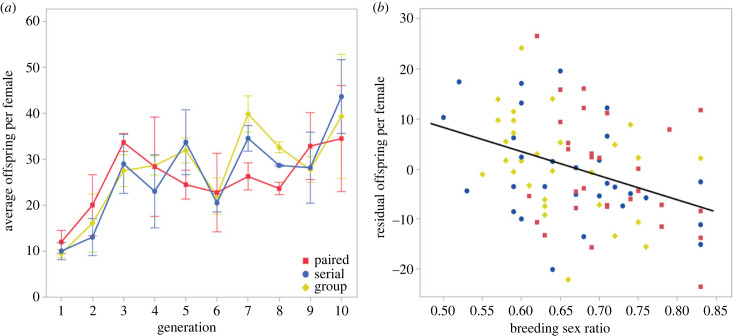
Effect of generation and sex ratio on average offspring produced per female. (*a*) Offspring production per female increased over the course of the experiment, (*b*) but decreased as sex ratio became female-biased. In (*b*), we use the residual of average offspring per female on generation to represent variation not explained by the increase over time (shown in *a*).

The dynamics of the drive chromosome also had pronounced demographic effects. Eclosion sex ratio exceeded the breeding sex ratio cap (83% female) nine times—five times in a paired treatment line, three times in a serial treatment line and one time in a group treatment line (electronic supplementary material, figure S3). In addition, the number of flies eclosing within each line changed as a function of generation and sex ratio. A LMM fit to offspring production per female in drive lines (table 2; electronic supplementary material, tables S5 and S6) showed that despite a three- to fivefold increase over the course of the experiment (figure 2*a*), offspring production per female declined as breeding sex ratio became increasingly female-biased (figure 2*b*). The random effect of line did not differ from zero (5.10 ± 8.64, *p* = 0.55). Analysis of only group-mated lines showed that the increase in offspring per female over time did not differ between lines initiated with and without drive (electronic supplementary material, tables S15–S17). A similar analysis of offspring production by line, rather than by female, revealed evidence of a quadratic relationship with breeding sex ratio such that maximum offspring production per line occurred when the sex ratio was about 70% female (electronic supplementary material, tables S18–S20 and figure S4).

**Table 2.** Results from a LMM on offspring per female in *drive* lines, where line is a random effect nested within treatment, and treatment, generation, and their interaction, as well as average female length and breeding sex ratio are fixed effects.

effect	offspring per female		
	d.f.	*F*	*p*
mating treatment	2, 6.2	0.15	0.87
generation	1, 78.7	22.35	<0.0001
treatment * generation	1, 77.0	0.31	0.74
average female length	1, 82.0	1.75	0.19
breeding sex ratio	1, 66.5	9.37	0.0032

**Figure 3.**
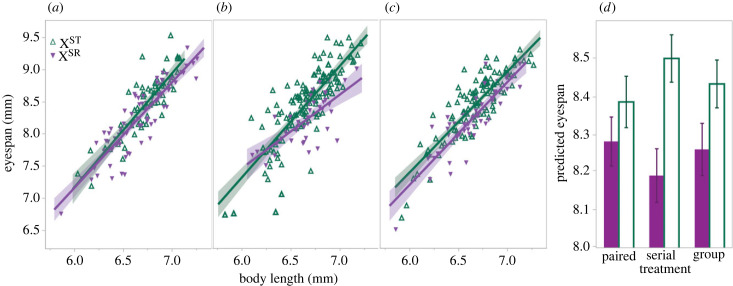
Male eyespan as a function of body size and X chromosome type in the (*a*) paired, (*b*) serial and (*c*) group with drive treatments at the end of the experiment (generations 10 and 11). *Post hoc* tests (*p* < 0.05) on relative male eyespan (*d*) show significant differences by X chromosome type and between X^ST^ and X^SR^ males from the serial versus each of the other mating treatments.

The LMM fit to male eyespan, a target of pre-copulatory sexual selection, in drive lines revealed effects of body length, X chromosome type, and interactions between mating treatment and X chromosome type and between body length and X chromosome type (table 3; electronic supplementary material, tables S7 and S8). Random effects did not differ from zero (line: 0.008 ± 0.005, *p* = 0.14; generation: 0.001 ± 0.002, *p* = 0.60). The effect of X chromosome type is due to X^ST^ males having relatively larger eyestalks than X^SR^ males, as expected, in all treatments. *Post hoc* tests on the interaction between treatment and X chromosome type reveal that relative eyespan in the serial treatment differed from the overall mean (figure 3); X^ST^ males in the serial treatment had longer eyestalks (*post hoc* ANOM, *t* = 2.69, *p* = 0.0424) while X^SR^ males of that treatment had shorter eyestalks (*post hoc* ANOM, *t* = −3.42, *p* = 0.0040). No other difference was detected for X^SR^ or X^ST^ males of the paired or group treatments. The comparison of relative eyespan in X^ST^ males from the group, drive treatment to the group, no-drive treatment detected no difference (electronic supplementary material, tables S21–S23). A similar LMM fit to female eyespan also detected a strong effect of body length (table 3; electronic supplementary material, tables S9 and S10), but no effect of X chromosome type, treatment or any interaction. LMMs of male and female body length showed no effect of treatment or X chromosome type in either sex (electronic supplementary material, tables S24–S29).

**Table 3.** Results from two LMMs in *drive* lines on relative male eyespan and relative female eyespan, with random effects of line nested within treatment and generation (10 or 11), and fixed effects of mating treatment, body length, X chromosome type and their interactions.

effect	male			female		
	d.f.	*F*	*p*	d.f.	*F*	*p*
treatment	2, 6.1	0.02	0.98	2, 6.7	0.03	0.97
body length	1, 447.8	856.53	<0.0001	1, 251.8	314.72	<0.0001
X type	1, 447.4	54.46	<0.0001	2, 251.4	2.29	0.10
treatment * body length	2, 441.4	2.19	0.11	2, 252.0	0.47	0.63
treatment * X type	2, 447.1	5.16	0.0061	4, 251.0	0.73	0.57
body length * X type	1, 444.1	4.83	0.0285	2, 248.2	0.13	0.88
treatment * body length * X type	2, 444.3	2.23	0.11	4, 248.5	0.22	0.93

**Figure 4.**
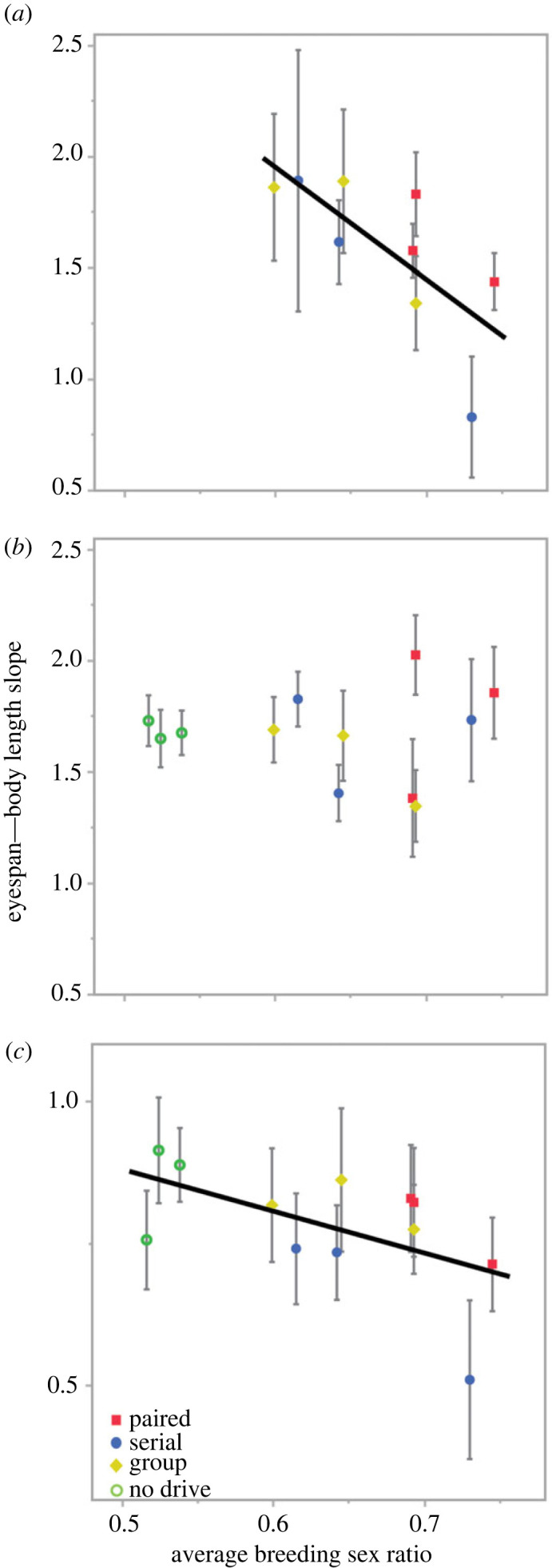
Slopes from OLS regressions of eyespan on body length (error of the slope) plotted against the average breeding sex ratio within each experimental line for (*a*) X^SR^ males, (*b*) X^ST^ males and (*c*) females at the end of the experiment in the four treatments. Eyespan allometric slope in X^SR^ males and females declines as the average breeding sex ratio increases, but does not change in X^ST^ males.

A linear model fit to eyespan allometric slopes from males in each line with treatment, average breeding sex ratio, X chromosome type and the interaction between breeding sex ratio and X chromosome type included as effects was significant (*F* = 3.08, *N* = 21, *p* = 0.0386; electronic supplementary material, tables S11 and S12). Male allometric slope did not differ by treatment (*F* = 2.14, d.f. = 3, *p* = 0.14) or X chromosome type (*F* = 0.16, d.f. = 1, *p* = 0.69) but was affected by average breeding sex ratio (*F* = 9.73, d.f. = 1, *p* = 0.0075) and by the interaction between breeding sex ratio and X chromosome type (*F* = 6.93, d.f. = 1, *p* = 0.02). The interaction is due to eyestalk allometric slope decreasing in X^SR^ males (figure 4*a*) as breeding sex ratio increased, but not in X^ST^ males (figure 4*b*). A similar linear model fit to female eyespan allometric slopes (*F* = 5.14, *N* = 12, *p* = 0.03; electronic supplementary material, tables S13 and S14) also showed evidence of the allometric slope decreasing when average breeding sex ratio increased (figure 4*c*; *F* = 6.64, d.f. = 1, *p* = 0.037), and a marginal effect of treatment (*F* = 4.07, d.f. = 3, *p* = 0.057).

## 4. Discussion

Experimental manipulation of female mating opportunities in *T. dalmanni* stalk-eyed flies reveals that multiple mating by females, with or without pre-copulatory choice, reduces the frequency of a driving X chromosome to levels similar to those observed in wild populations. The frequency of X^SR^ chromosomes and the sex ratio at eclosion oscillated as X chromosomes were passed from affected males to carrier daughters and then back to affected sons; however, the overall pattern supports an important role for sexual selection in limiting the spread of drive in this species. As expected, the frequency of the X^SR^ chromosome and the eclosion sex ratio rapidly increased in the paired treatment lines, some of which would have gone extinct if we had not imposed a limit on the breeding sex ratio. Interestingly, the serial and group treatments controlled X^SR^ frequency to a similar extent despite the known genetic association between X chromosome type and male eyespan [13,24]. These results are consistent with a previous experimental evolution study which removed sexual selection and found that multiple mating in female *D. pseudoobscura* was sufficient to limit the sex ratio phenotype and prevent line extinction [43,44].

Offspring production increased over the course of the experiment in all lines. The number of offspring produced by the lines reached a maximum at a breeding sex ratio of approximately 70% but was lower at higher sex ratios, consistent with expectations [1,5]. The per female increase in offspring production over time is not explained by changes in average female body length or the presence of drive and so must have some other cause, such as inadvertent selection for rapid egg laying or more efficient fertilization. Nevertheless, a negative effect of female-biased sex ratios on offspring production per female is consistent with sperm limitation: as drive became more common in a line, females had access to fewer males while each male could mate with more females per day. When the breeding sex ratio was female-biased, we suspect that females received and stored fewer sperm and consequently produced fewer offspring. This result is consistent with a previous study that showed a fertilization deficit when a male, of either X chromosome type, was presented with five mates instead of one [45]. Differences in fecundity between female drive genotypes might contribute to this effect, but we think that is less likely given that a previous study found a heterozygote advantage for female fecundity [30].

Experimental evolution also reveals that selection can affect male eyespan, a sexually selected allometric trait, in multiple ways. Given that the X^SR^ chromosome is known to have a strong effect on male eyespan [13,24], we compared relative eyespan by chromosome type in each mating treatment to avoid confounding eyespan change due solely to change in X^SR^ frequency. We expected that reduced intensity of pre-copulatory sexual selection imposed by the paired and serial treatments would result in reduced male eyespan compared to the group treatment; however, we did not observe that outcome. A potential explanation for this result is that the relatively high density of flies in the group mating cages prevented males from controlling access to females or females from exerting choice. Finding that only male relative eyespan in the serial treatment differed from other drive treatments is more difficult to explain, but could be the result of correlated change due to linkage between X-linked genes that influence male or female traits involved in sperm competition [29,46,47]. The difference in relative eyespan between males in the serial treatment and males in the other treatments is small when compared to the outcome of direct artificial sexual selection [39], which is consistent with relatively weak cumulative selection imposed by manipulating mating opportunities and correlated change due to linkage.

Analysis of allometric slopes in both sexes indicates that the population dynamics of the X^SR^ chromosome may also have morphological consequences, as changes in the breeding sex ratio likely influence the intensity of selection experienced in each line. A line with a more female-biased sex ratio should have reduced sexual selection on male traits, as males face less competition and otherwise less successful X^SR^ males will be more likely to gain fertilizations. Sexual selection favours positive allometry in the male ornament [48], so it is not surprising that lines with female-biased breeding sex ratios are associated with decreased allometry, as observed in X^SR^ males and females (figure 4). Overall, these analyses indicate that eyespan in these flies can be impacted by the dynamics of the X^SR^ chromosome and that, in males, each X chromosome type experiences selection differently despite a shared environment and autosomal background.

By manipulating the mating environment, in this study, we used experimental evolution to reveal potential interactions between sexual selection and meiotic drive. While largely consistent with expectations for how multiple mating can control the spread of a drive element [4], our results also reveal that an X-linked distorter can have varied impacts on the evolution of sexually selected traits. These findings suggest that the response to selection of X-linked male traits in *T. dalmanni* depends on selection intensity, which varies with the eclosion sex ratio due to the frequency of the X^SR^ chromosome. In addition, the two types of X chromosomes respond to selection differently, perhaps due to differences in selection intensity, effective population size and genetic variation [32]. Despite these differences, divergence is constrained because all X-linked genes must maintain functionality with the rest of the genome. Furthermore, the evolution of sexually selected traits in the presence of drive is likely of greater complexity than described here, as our analysis does not consider any coevolutionary changes that may arise if female mating behaviour or post-copulatory traits also respond to the frequency of drive, as may be expected [15]. Given that many sex-linked distorters are associated with one or more chromosomal inversions [2] and are impacted by sexual selection [4], similar dynamics seem likely to influence the evolution of other natural drive systems and could have relevance for the application of synthetic drive systems in wild populations where sexual selection may influence both the persistence of the driver and phenotypic evolution.

## Data Availability

Phenotype and genotype data are available from the Dryad Digital Repository: https://doi.org/10.5061/dryad.r2280gbj3 [49]. Additional information is provided in the electronic supplementary material [50].
